# Trends in avoidable mortality from cardiovascular diseases in the European Union, 1995–2020: a retrospective secondary data analysis

**DOI:** 10.1016/j.lanepe.2024.101079

**Published:** 2024-09-27

**Authors:** Avi Cherla, Ilias Kyriopoulos, Pauline Pearcy, Zoi Tsangalidou, Haris Hajrulahovic, Pavlos Theodorakis, Charlotte E. Andersson, Mandeep R. Mehra, Elias Mossialos

**Affiliations:** a Department of Health Policy, London School of Economics and Political Science, London, UK; b Department of Statistics, Oxford University, Oxford, UK; c World Health Organization, Regional Office for Europe, Copenhagen, Denmark; d Department of Medicine, Brigham and Women's Hospital and Harvard Medical School, Boston, MA, USA

**Keywords:** Cardiovascular disease, Avoidable mortality, Europe

## Abstract

**Background:**

Certain causes of death can be avoided with access to timely prevention and treatment. We quantified trends in avoidable deaths from cardiovascular diseases for European Union (EU) countries from 1995 to 2020 and examined variations by demographics, disease characteristics, and geography.

**Methods:**

Retrospective secondary data analysis of avoidable cardiovascular mortality using the WHO Mortality Database. Avoidable causes of death were identified from the OECD and Eurostat list (which uses an age threshold of 75 years). Regression models were used to identify changes in the trends of age-standardized mortality rates and potential years of life lost.

**Findings:**

From 1995 to 2020, 11.4 million deaths from cardiovascular diseases in Europe were avoidable, resulting in 213.1 million potential life years lost. Avoidable deaths were highest among males (7.5 million), adults 65–74 years (6.8 million), and with the leading cause of death being ischemic heart disease (6.1 million). From its peak in 1995 until 2020, avoidable mortality from cardiovascular diseases has decreased by 57% across the EU. The difference in avoidable cardiovascular diseases mortality between females and males, and between Eastern and Western Europe has reduced greatly, however gaps continue to persist.

**Interpretation:**

Avoidable mortality from cardiovascular diseases has decreased substantially among EU countries, although improvement has not been uniform across diseases, demographic groups or regions. These trends suggest additional policy interventions are needed to ensure that improvements in mortality are continued.

**Funding:**

10.13039/100004423World Health Organization, Regional Office for Europe.

**Research in context**

**Evidence before this study**

Avoidable mortality quantifies the number of deaths that could be avoided through effective public health and primary prevention strategies, and access to timely and effective medical care. The causes of death that are considered avoidable are informed by research while the age threshold is set at 75 years—the lowest life expectancy across OECD and European countries. We searched PubMed and Google Scholar from database inception until February 1, 2023, for research that has quantified trends in avoidable cardiovascular mortality across European countries, using the search terms “avoidable mortality”, “cardiovascular disease, “Europe”, and “European Union”. There have been a number of studies which have provided country level estimates for avoidable cardiovascular mortality. The most comprehensive evaluation across European countries was a study which focused on avoidable deaths from ischemic heart disease, heart failure, and hypertension from 1978 to 2000, and the associated effects of certain health interventions on reductions in avoidable deaths.

**Added value of this study**

This study quantified trends in avoidable mortality from cardiovascular disease for European Union (EU) countries from 1995 to 2020. Using cross-sectional mortality data from the World Health Organization for every country, we found that more than 11 million deaths and 213 million life years lost in Europe from cardiovascular disease were from avoidable causes. Nearly 2 of every 3 deaths were in males and 2 of every 5 deaths were in working age adults (25–64 years). The difference in avoidable cardiovascular diseases mortality between females and males, and between Eastern and Western Europe has reduced greatly, however gaps continue to persist.

**Implications of all the available evidence**

Reductions in avoidable mortality from cardiovascular disease are heterogenous, with key differences by age, sex, and country. The reasons for these differences are multifaceted and require further attention to behavioural, economic, and social factors, including public health and healthcare systems.

## Introduction

Cardiovascular disease is the leading cause of death and disability in Europe.^1^ Nearly one-third of all deaths in Europe annually (1.7 million) are attributed to cardiovascular diseases.^2^^,^^3^ The associated costs from health and long-term care, productivity losses, and informal care from cardiovascular diseases amount to €282 billion annually.^4^ Estimates project that the public health burden and economic costs associated with cardiovascular disease will likely increase in the coming years as many high-income countries experience a plateau or reversal in progress with cardiovascular mortality.^1^^,^^5^, ^6^, ^7^ Stalled progress with cardiovascular disease prevention and treatment has contributed to a recent leveling-off in life expectancy for Europeans.^8^

It is important to understand the degree to which deaths from cardiovascular disease could be avoided through effective public health and primary prevention strategies, and access to timely and effective medical care. Originally proposed in 1976 as a quality measure for medical care, avoidable mortality quantifies unnecessary and untimely deaths that are considered preventable or treatable.^9^ The causes of death that are considered avoidable are informed by research while the age threshold is set at 75 years—the lowest life expectancy across OECD and European countries.^10^ For example, ischemic heart disease and cerebrovascular disease are the two leading causes of cardiovascular mortality in Europe and can both be avoided through effective prevention and treatment.^11^, ^12^, ^13^

Disparities across Europe in cardiovascular disease incidence, treatment, and outcomes are an area of much research.^3^^,^^14^, ^15^, ^16^, ^17^, ^18^, ^19^, ^20^ For example, some studies provide country level estimates of avoidable cardiovascular mortality.^12^^,^^13^^,^^21^, ^22^, ^23^, ^24^, ^25^, ^26^ The most comprehensive evaluation across European countries focused on avoidable deaths from ischemic heart disease, heart failure, and hypertension from 1978 to 2000, and the associated effects of certain health interventions on reductions in deaths.^21^ It is important to understand how the trajectory of avoidable mortality from cardiovascular disease compares across European countries in recent years. At a population level, this data can serve as an indicator for informing public policy and investments in European health systems.

In this study, we quantified trends in avoidable mortality from cardiovascular diseases for European Union (EU) countries from 1995 to 2020 and how these varied by demographics, disease characteristics, and geography.

## Methods

### Data

We accessed cause, age, and sex specific data on deaths from the World Health Organization (WHO) Mortality Database, a compilation of data from national vital registration systems. Data is updated annually based on voluntary reporting from countries and is limited to medically certified causes of mortality according to the International Classification of Diseases (ICD) codes. Individual-level characteristics beyond age and sex (e.g., race) are not reported.

The time frame for our analysis was informed by data availability from the WHO Mortality Database (updated on 21 February 2024). The WHO Mortality Database is subject to a lag in reporting, as countries generally submit their mortality data 12–18 months after the closure of their records for the calendar year. At the time of our analysis, 2020 was the latest year for which most countries had mortality data available (the supplement provides information on data availability for each country). We selected 1995 as the starting year owing to data reliability and consistency issues prior to this. Therefore, we focused on EU countries from 1995 to 2020, during the majority of which time the United Kingdom was a member. As 1995 predated the introduction of ICD-10 mortality reporting for several countries, we harmonized data using ICD-9 to ICD-10 mapping.^27^^,^^28^

Because data from the WHO Mortality Database are subject to voluntary reporting from countries, delays in publishing data or gaps in reporting may occur.^5^^,^^16^^,^^29^^,^^30^ Over the 26 year analysis period, 2.6% (19/728; 28 countries over 26 years) of country-year observations were unavailable (Supplement Table S1). As gaps in reporting are largely concentrated in the terminal years, several statistics are presented using the earliest and latest years of data available for each country.

In a sensitivity analysis to estimate the effect of missing data on our findings, we imputed missing values using the last observation carried forward^29^ and linear interpolation. The findings were consistent with the primary analysis (within 1–2% of the average age standardized mortality rates) and are included in Supplement Table S3.

### Avoidable causes of death

Avoidable mortality refers to premature deaths below 75 years of age that could have been avoided through effective public health and primary prevention strategies or timely and appropriate treatment.^11^ We used a jointly determined list published by the OECD and Eurostat in 2022 to identify avoidable (preventable and treatable) causes of death.^10^ This list harmonizes earlier research on quantifying avoidable mortality and has been widely cited in the published literature.^12^^,^^23^^,^^31^^,^^32^ It uses 75 years as the age threshold for premature death because this is the lowest life expectancy across OECD and European countries.

The OECD/Eurostat list includes an allocation of avoidable mortality for each disease that is considered preventable or treatable. For example, ischemic heart disease, one of the leading causes of cardiovascular mortality, is considered 50% preventable and 50% treatable. This allocation was determined due to the relatively equal contributions of prevention and treatment for reducing mortality from ischemic heart disease. In contrast, rheumatic and other heart diseases are considered 100% treatable. For all diseases included in this analysis and for all premature deaths, the proportional allocation of preventable and treatable mortality sum to 100%. Table 1 includes the OECD/Eurostat estimate for contribution of deaths attributable to prevention and treatment for each disease.

**Table 1:** Avoidable causes of death from cardiovascular diseases.

Cause of death	ICD-10 code	Diseases included	Preventable	Treatable
Aortic aneurysm	I71	Aortic aneurysm and dissection	50%	50%
Hypertensive diseases	I10–I13, I15	Primary hypertension; Hypertensive heart disease; Hypertensive renal disease; Hypertensive heart and renal disease; Secondary hypertension	50%	50%
Ischemic heart diseases	I20–I25	Angina pectoris; Acute myocardial infarction; Subsequent myocardial infarction; Other acute ischemic heart diseases; Chronic ischemic heart disease	50%	50%
Cerebrovascular diseases	I60–I69	Subarachnoid hemorrhage; Intracerebral hemorrhage; Other nontraumatic intracranial hemorrhage; Cerebral infarction; Stroke (not specified as hemorrhage or infarction); Other cerebrovascular diseases; Sequelae of cerebrovascular disease	50%	50%
Other atherosclerosis	I70, I73.9	Atherosclerosis; Peripheral vascular disease (unspecified)	50%	50%
Rheumatic and other heart disease	I00–I09	Rheumatic fever without mention of heart involvement; Rheumatic fever with heart involvement; Rheumatic chorea; Rheumatic mitral valve diseases; Rheumatic aortic valve diseases; Rheumatic tricuspid valve diseases; Multiple valve diseases; Other rheumatic heart diseases		100%
Venous thromboembolism	I26, I80, I82.9	Pulmonary embolism; Phlebitis and thrombophlebitis; Embolism and thrombosis of unspecified vein		100%

Notes: Preventable and treatable percentage allocations refer to mortality in those aged 0–74 years of age. For venous thromboembolism, the OECD and Eurostat report notes that: *“conditions that are mainly acquired when people are hospitalized or in contact with health services might also be considered to be preventable, in the sense that the incidence of these health care associated infections or health problems might be reduced through greater prevention in health care facilities.”*.

We focused on avoidable “diseases of the circulatory system” (ICD-10 code) from the OECD/Eurostat list: aortic aneurysm (I71), hypertensive diseases (I10–I13, I15), ischemic heart diseases (I20–I25), cerebrovascular diseases (I60–I69), other atherosclerosis (I70, I73.9), rheumatic and other heart disease (I00–I09), and venous thromboembolism (I26, I80, I82.9).

Ill-defined causes of death (R00–R99) was not specified in the list of avoidable causes of mortality and accounted for less than 0.031% of the cardiovascular mortality records. In a sensitivity analysis we reallocated unspecified mortality records using 1) a proportional reallocation of unspecified mortality, and 2) a redistribution of unspecified mortality using methods from the Global Burden of Disease studies.^33^ The results (age-standardized mortality estimates and country ranking) were consistent with the primary analysis.

### Potential life years lost

We next estimated the life years lost from avoidable cardiovascular deaths. This was done by comparing the age at death to the remaining life expectancy for an individual with the same demographic and geographic profile, resulting in the years of life lost from premature death. Avoidable deaths which occur at younger ages result in more life years lost relative to deaths which are closer to the full life expectancy.

We quantified the avoidable life years lost from cardiovascular diseases based on standardized life tables produced by the Global Burden of Disease.^34^^,^^35^ Life tables report the yearly life expectancy estimates for each country in five-year age ranges. While other international agencies (WHO, OECD, and Eurostat) also produce life tables, the life tables produced by the 2019 Global Burden of Disease study most closely align with the WHO Mortality Database reporting in terms of frequency, coverage, and granularity.

We matched aggregated death counts with the corresponding remaining life expectancy based on the country, year, sex, and age at death. We then calculated the potential life years lost by multiplying deaths with the remaining years of life expectancy for each subgroup. Since life tables were only available through 2019, we calculated the potential years of life lost until then.

### Statistical analysis

Consistent with population health research, we calculated age-standardized mortality rate estimates per 100,000 individuals, using the European standard population distribution (a direct age-adjusted standardization).^36^ The age-standardized mortality rate is a weighted average of age-specific mortality rates, where weights are applied based on the proportional representation of each age group within the European standard population. The standardization accounts for differences in the population age structure across countries and over time.

We stratified mortality estimates and potential life years lost by cardiovascular disease subtype, preventable vs treatable causes of death, country, sex, and age. Countries were further grouped into regions consisting of Eastern and Western Europe, based on sociopolitical and historical factors. Eastern European countries included the former European Eastern Bloc (Bulgaria, Czech Republic, Hungary, Poland, Romania, and Slovakia); former Soviet Union (Estonia, Latvia, and Lithuania); and former Yugoslavia (Croatia and Slovenia) (eTable 1). Western Europe is defined as the remaining countries. Age was categorized into three discrete buckets: under 25, 25–64 (referred to as working age), and 65–74 (since 75 is the upper threshold for defining premature deaths). We summarized estimates according to working age (25–64) and non-working age (under 25, 65–74) populations to quantify the economic impact of avoidable mortality on the working population.

Trends were modelled using the Joinpoint regression software (developed by the US National Cancer Institute) and presented as average annual percentage changes.^37^ Joinpoint regressions segment trend data and are used to identify and quantify break points in linear time series data. Break points are determined based on multiple linear trends estimated across a period of observations. All Joinpoint regressions were univariable pooled analyses. Models were selected using the minimized Bayesian information criterion. Statistical analyses were performed using Python (version 3.9.12).

### Ethical clearance

No ethical clearance was needed as this study used secondary data sources.

### Role of the funding source

The funding source was not involved in the data collection, analysis, or writing of this research. However, two coauthors from the funder were involved in the interpretation of data and revision of the manuscript.

## Results

### Avoidable cardiovascular mortality

From 1995 to 2020, an estimated 11.4 million deaths from cardiovascular diseases in Europe were attributed to avoidable causes. Two of every three (66.3%, 7.5 million) avoidable deaths were in males. By age group, the post-working age population (65–74 years) was the leading contributor of avoidable cardiovascular mortality (60.2%, 6.8 million) while working age adults (25–64 years) accounted for 39.6% (4.5 million) of deaths. The under 25 population made up a nominal proportion of the avoidable cardiovascular mortality.

Slightly more deaths from cardiovascular disease were considered treatable (52.3%, 5.9 million) than preventable (47.7%, 5.4 million). Ischemic heart disease followed by cerebrovascular disease and hypertensive diseases were the leading causes of avoidable cardiovascular mortality. Together they accounted for 88.8% (10.1 million) of avoidable cardiovascular deaths. Table 2 further summarizes the cumulative estimates from 1995 to 2020.

**Table 2:** Cumulative avoidable mortality and potential years of life lost, 1995–2020.

Characteristics	Mortality	Potential life years lost
Avoidable	11,350,741	213,114,679
Preventable	5,413,119 (47.7%)	100,917,300 (47.4%)
Treatable	5,937,622 (52.3%)	112,197,379 (52.6%)
Sex		
Male	7,527,997 (66.3%)	136,810,878 (64.2%)
Female	3,822,744 (33.7%)	76,303,801 (35.8%)
Age		
0–9	4423 (0.0%)	329,799 (0.2%)
10–19	7616 (0.1%)	478,764 (0.2%)
20–29	35,002 (0.3%)	1,853,003 (0.9%)
30–39	155,575 (1.4%)	6,658,075 (3.1%)
40–49	675,792 (6.0%)	22,638,728 (10.6%)
50–59	1,871,585 (16.5%)	47,023,330 (22.1%)
60–69	4,477,339 (39.4%)	79,424,486 (37.3%)
70–74	4,123,409 (36.3%)	54,708,494 (25.7%)
Disease		
Aortic aneurysm	332,959 (2.9%)	6,434,722 (3.0%)
Cerebrovascular disease	3,176,282 (28.0%)	59,389,057 (27.9%)
Hypertensive disease	767,090 (6.8%)	13,730,955 (6.4%)
Ischemic heart disease	6,135,217 (54.1%)	115,691,653 (54.3%)
Other atherosclerosis	414,689 (3.7%)	6,588,213 (3.1%)
Rheumatic and other heart disease	124,365 (1.1%)	2,561,997 (1.2%)
Venous thromboembolism	400,139 (3.5%)	8,718,082 (4.1%)
Region		
Eastern Europe	4,769,292 (42.0%)	81,121,120 (38.1%)
Western Europe	6,581,449 (58.0%)	131,993,559 (61.9%)

Values are cumulative figures and are not presented as age standardized mortality rates. Within each subgrouping included above, percentages represent share of total mortality or potential years of life lost. Percentages may not sum to 100% due to rounding.

### Trends in demographic and disease characteristics

**Fig. 1:**
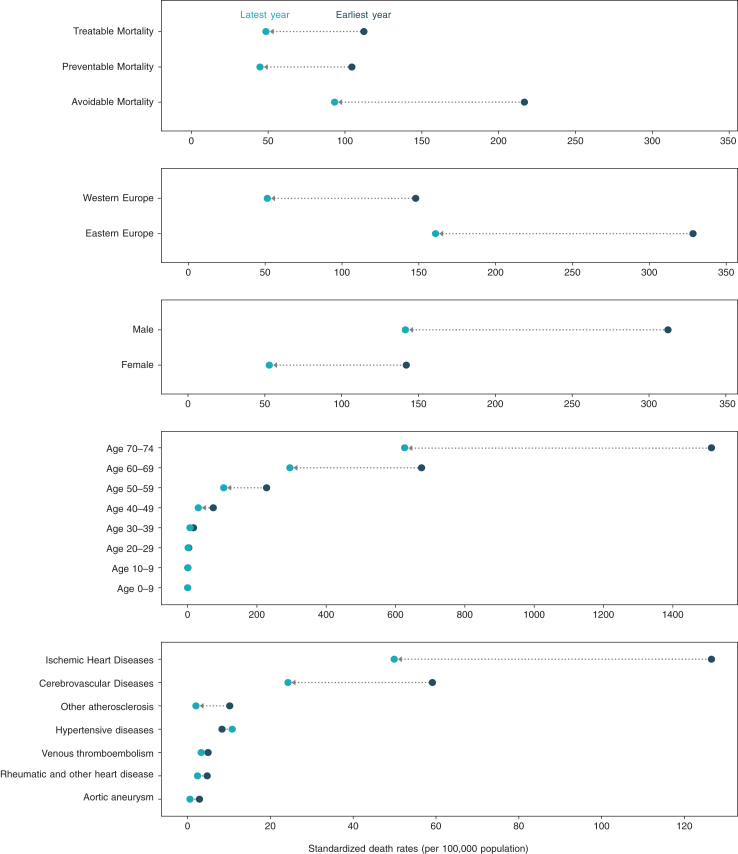
**Changes in avoidable cardiovascular mortality rate from earliest to latest year, by demographic features and disease.** x-axis scales vary across graphs.

**Fig. 2:**
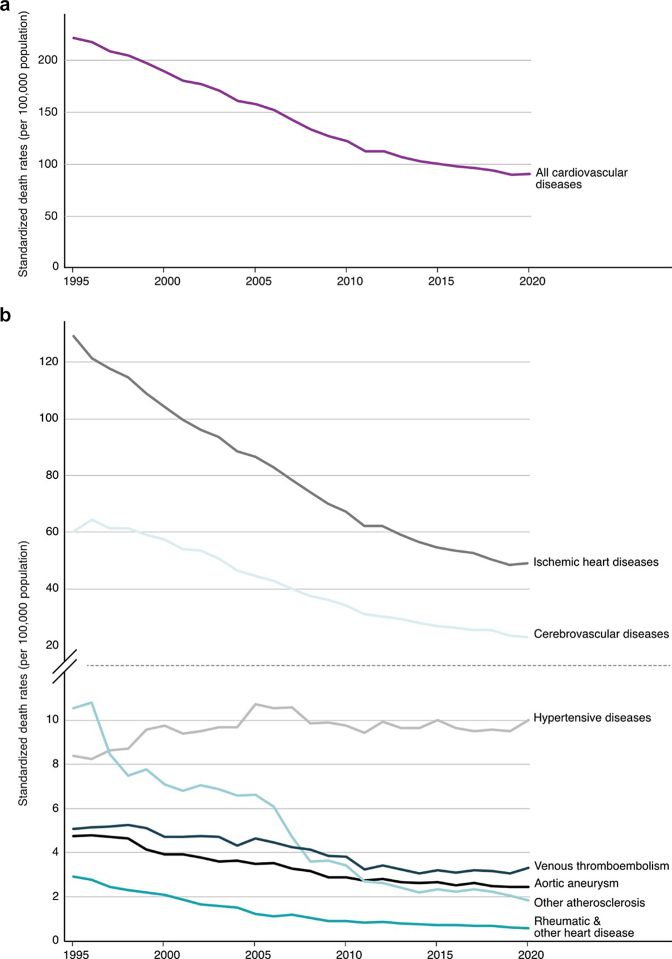
**Trends in avoidable cardiovascular mortality (a) and by disease (b).** Results from the joinpoint regression are detailed in the Supplement.

Avoidable mortality from cardiovascular disease decreased from a peak of 216.7 per 100,000 in the earliest year of data to 93.2 per 100,000 by the latest year of data (Figs. 1 and 2a). Stratified by key demographics, males had a greater reduction in avoidable deaths (312.3–141.4 per 100,000) compared to females (142.0 per 100,000–52.9 per 100,000). Reductions in mortality in the post-working age population (age 65–74) (1172.1–492.0 per 100,000) were also greater than in the working age population (age 25–64) (139.4–62.4 per 100,000).

The reduction in age-standardized mortality rates for treatable causes (112.3–48.6 per 100,000) and preventable causes (104.5–44.7 per 100,000) was similar. Fig. 1 shows that ischemic heart disease (126.5–49.9 per 100,000) and cerebrovascular disease (59.1–24.3 per 100,000) had the greatest reductions in mortality. Conversely, there was a slight increase in mortality for hypertensive diseases (8.3–10.8 per 100,000).

There was a substantial reduction in avoidable mortality for all causes starting in the early 2000's. However, more recent trends for several diseases show that there has been a deceleration in the rate of decline relative to previous years (average annual percentage change: aortic aneurysm −1.50% [95% CI: −1.69%, −1.30%], cerebrovascular disease −3.26% [95% CI: −3.69%, −2.68%], ischemic heart diseases −3.04% [95% CI: −3.32%,−2.62%], other atherosclerosis −3.26% [95% CI: −4.74%,−1.42%], rheumatic and other heart diseases −9.26% [95% CI: −13.14%, −3.97%]). Trends in avoidable mortality for some diseases has plateaued or increased relative to previous years (hypertensive diseases −0.05% [95% CI: −0.92%, 1.09%], venous thromboembolism 0.16% [95% CI: −0.78%, 1.49%]), although the difference was not significant. (Fig. 2b, Supplement Figure S2).

### Geographical differences

Pooled across all years, avoidable cardiovascular mortality rates were greater in Eastern European countries compared to Western Europe (233.1 vs 85.7 per 100,000 annually). Similar differences were observed for preventable mortality (112.6 and 41.0 per 100,000 annually) and treatable mortality (120.5 and 44.7 per 100,000 annually). Comparing trends across the two regions from 1995 to 2020, Eastern European countries had a greater reduction (329.9–158.0 per 100,000) in avoidable cardiovascular deaths compared to Western European countries (143.5–51.3 per 100,000) (Fig. 1).

**Fig. 3:**
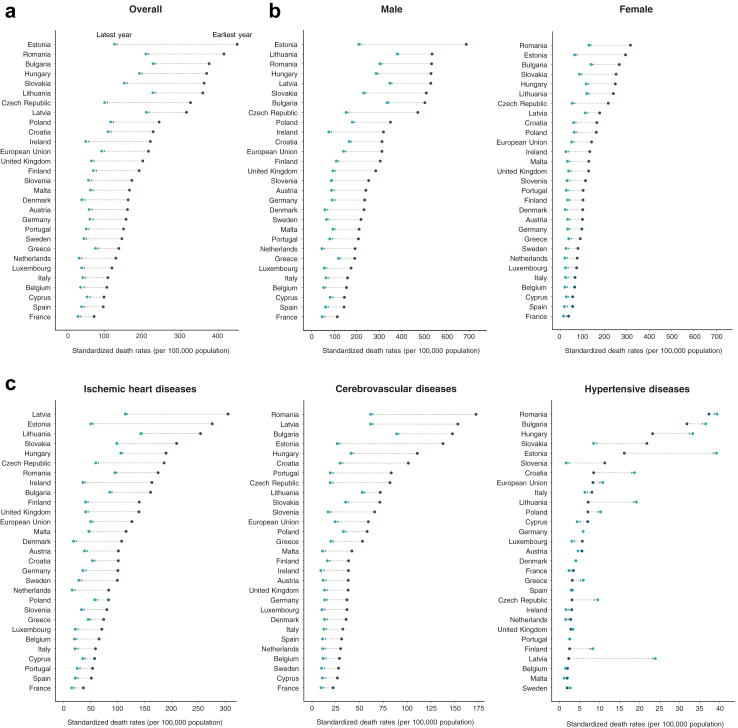
**Change in avoidable cardiovascular mortality per country (a) overall (b) by sex and (c) from the top 3 causes of death.** x-axis scales vary across graphs.

Fig. 3a shows the changes in mortality for each country from the earliest to latest years of data. Latvia, Romania, and Bulgaria had the highest average age-standardized mortality rates, while France, Spain, and Italy had the lowest. There was over a sixfold difference between Latvia (316.8 per 100,000), the country with the highest avoidable mortality rate, and France (46.4 per 100,000), the country with the lowest.

For many causes, avoidable mortality was more than twice the rate in Eastern Europe compared to Western Europe. These include cerebrovascular disease (69.9 vs 22.1 per 100,000), hypertensive disease (19.0 vs 3.5 per 100,000), ischemic heart disease (122.7 vs 51.5 per 100,000), other atherosclerosis (10.5 vs 1.3 per 100,000), and rheumatic and other heart disease (2.2 vs 0.7 per 100,000) (Fig. 3c).

Stratified by sex, males in Eastern Europe had more than twice the rate of avoidable mortality as males in Western Europe (345.3 vs 124.5 per 100,000), as did females (148.5 vs 51.0 per 100,000) (Fig. 3b). For females, the difference in age-standardized mortality rates due to cerebrovascular diseases (3.0×), hypertensive diseases (5.4×), and other atherosclerosis (8.0×) were even more pronounced between the two regions. Comparatively for males, mortality rates due to cerebrovascular disease (3.4×), hypertensive diseases (5.8×), other atherosclerosis (8.4×), and rheumatic and other heart disease (3.7×) had the largest discrepancies between regions.

The difference in avoidable mortality rates between Eastern and Western Europe was observed across all ages. Among working-age adults, cerebrovascular disease (3.3×), hypertensive disease (6.0×), ischemic heart disease (2.5×), other atherosclerosis (8.2×), and rheumatic and other heart disease (4.3×) were all over 2× as prevalent in Eastern Europe compared to Western Europe. In the non-working age population, aortic aneurysm was the only disease with a higher age-standardized mortality rate in Western European countries.

### Potential life years lost

Avoidable cardiovascular deaths resulted in 213.1 million potential life years lost for EU countries from 1995 to 2019 (1859 per 100,000 annually). Table 2 summarizes the cumulative life years lost according to key characteristics. The rate of potential life years lost was nearly twice as high in males as in females, accounting for an average 5.5 million potential life years lost annually in males and 3.1 million in females. By age group, 4.5 million potential life years were lost on average annually in working age adults (25–64 years of age).

Comparing trends in the potential life years lost since 1995, Supplement Figure S3 shows that males have had a greater reduction compared to females. More potential life years were lost from treatable than preventable causes, representing an annual average 4.5 million potential life years lost for treatable causes, and 4.0 million for preventable causes. By disease, potential years of life lost were highest due to ischemic heart disease (1009 per 100,000 annually) and cerebrovascular diseases (518 per 100,000 annually). For most diseases, there was considerable progress in reducing potential years of life lost over the study period. However, for hypertensive diseases the potential life years lost increased over the course of the study period (104.1–129.9 per 100,000).

Potential years of life lost were more than double in Eastern European countries Western Europe (3248 vs 1474 per 100,000 annually). From 1995 to 2019, there have been improvements in potential years of life lost for Eastern European countries, but these have not matched the level of Western Europe (Supplement Figure S3).

## Discussion

From 1995 to 2020, more than 11 million deaths and 213 million life years lost in Europe due to cardiovascular disease were from avoidable causes. Although avoidable cardiovascular deaths have more than halved since 1995, declines in mortality have not been consistent across diseases with persistent gaps between females and males, particularly in Eastern Europe. The sensitivity of these findings (mortality, life years lost, and country ranking) remained robust across several additional analyses that adjusted for ill-defined causes of mortality and missing data.

The trajectory of cardiovascular disease (incidence and prevalence) across Europe has remained stable or slightly decreased over time,^3^^,^^16^^,^^19^^,^^20^ suggesting that improvements in prevention and treatment have contributed to the reduction in avoidable cardiovascular mortality over the last several decades.^38^, ^39^, ^40^ Much greater attention than before is given to reducing risk factors by engaging in positive health behaviours such as physical activity, reducing dietary consumption of trans fats, and cessation of smoking and alcohol.^1^^,^^41^ Prescription drugs and surgical interventions have also played a large role in reducing avoidable deaths.^42^, ^43^, ^44^, ^45^

However, improvements across Europe are heterogeneously distributed. The persistent inequality in mortality between males and females suggests that more can be done to improve outcomes both in terms of prevention and treatment. The reasons for these differences are multifaceted, with many economic and social causes.^18^^,^^46^, ^47^, ^48^ For example, some observational evidence suggests that males are more likely to engage in risky health behaviours such as smoking and alcohol use.^49^ A 2022 Eurostat report noted that males were far more likely than females to be smokers, and the gap between males and females was the largest in Latvia and Romania, two countries with some of the highest annual average age-standardized mortality rates from avoidable cardiovascular mortality.^50^ However, some studies find contrary evidence.^18^^,^^47^ Ultimately more research is needed to understand and address the disparity between sexes. This research will become increasingly pertinent as obesity rates continue to rise, and electronic smoking and vaping products continue to gain popularity in the EU.

Avoidable deaths from cardiovascular disease also varied widely by geography. There was over a 6× difference in avoidable mortality rates between some European countries. These findings are consistent with previous studies and show that despite large reductions in mortality in Eastern Europe, mortality has not yet converged with the level of Western Europe.^13^, ^14^, ^15^, ^16^^,^^18^^,^^19^^,^^24^^,^^51^ This can be attributed to variable lifestyle factors and public policies, but also the quality of medical care and health systems, cost (out-of-pocket payments), and access to healthcare and prescription drugs that are consistently worse in Eastern Europe.^14^^,^^15^^,^^29^^,^^30^^,^^52^^,^^53^ These factors also contribute to non-adherence with treatment.^54^ In our study, avoidable mortality and potential years of life lost from cardiovascular disease were more than double the rate in Eastern Europe compared to Western Europe.

Targeted policies at both the national and regional level can help to further reduce deaths from cardiovascular disease in Europe. Since avoidable deaths reflect a combination of prevention and treatment, strategies for both are necessary. Research shows that modifiable risk factors contribute to 90% of the incident cases of cardiovascular disease.^1^^,^^55^ In 2021 the risk factors with the highest attributable burden for cardiovascular disease globally included high systolic blood pressure (10.8 million deaths), diet (6.6 million deaths), high levels of LDL cholesterol (3.8 million deaths), pollution (3.1 million deaths), and smoking (2.4 million deaths).^55^

For metabolic factors such as blood pressure and LDL cholesterol, policies that support early diagnosis and treatment are essential. Prescription drugs such as lipid-lowering agents, β-blockers, and antithrombotic agents can be highly effective if adherence is maintained.^38^^,^^42^^,^^54^ Another strategy to control metabolic as well as behavioural risk factors includes lifestyle changes: physical activity, improvements in nutrition, and cessation of alcohol and smoking. The additive effect of combining behaviour changes with drugs and other forms of treatment can be substantial.^39^^,^^56^

Policy incentives (and disincentives) can also be useful for encouraging behaviours. For example, additional taxes on tobacco products or ultra-processed foods to discourage consumption. However, designing policies that sustain behaviour changes is difficult.^57^, ^58^, ^59^ Finally, stricter regulations on fine particulate matter, pollution, and pesticides can help reduce exposure to environmental risk factors across the EU.^60^ To support further reductions in avoidable cardiovascular mortality, policymakers at the EU and Member State level should focus on policies which target the key metabolic, behavioural, and environmental risk factors which contribute to cardiovascular disease.

Our study has limitations. First, because we compare avoidable mortality across countries, there is a possibility that different countries have different diagnostic patterns, processes for death certification, and death coding.^11^^,^^61^ Assigning a death to a single cause can be subject to variation and may not accurately reflect patients with multiple comorbidities or cases where cardiovascular illness is a secondary cause of death. Some research suggests that these challenges may disproportionately impact females as they have lower rates of diagnosis.^62^

Second, since the WHO Mortality database is subject to reporting from member countries, there may be inconsistencies between countries with the quality, accuracy, and completeness of data. Regarding quality, the WHO provides a data usability rating for each country which reflects the completeness and proportion of ill-defined or non-specific causes. For 24/28 (86%) countries in our analysis the data usability was high (only Bulgaria had low data usability). See Supplemental Table S1 for a full list of data useability ratings by country. Another limitation is that member countries may be delayed in reporting, or may not consistently report data, which could systematically affect our results. In our study, 2.6% (19/728 country-years) of observations were missing or excluded for data reliability reasons. In sensitivity analyses reported in the supplement, we used different approaches for imputing missing data and obtained largely similar results, which lends support to our findings.

Third, the use of avoidable mortality as a health system performance measure has limitations. Some of the criticisms reflect the threshold of 75 years for premature deaths, the selection of conditions for which deaths should be ‘avoided’, the inability to account for the changing prevalence or incidence of diseases, or changes in how medical care is delivered.^11^ Nevertheless, avoidable mortality is a widely used indicator to identify further areas for research and improvement, specifically at the population level. It is also useful for international comparisons to identify areas where countries may be underperforming relative to their peers, as it is often difficult to find homogenous data at the country level for performance assessment.

### Conclusions

From 1995 to 2020, more than 11 million deaths and 213 million life years lost in Europe from cardiovascular disease were avoidable. Policymakers in Europe should prioritize health policies which target the key metabolic, behavioural, and environmental risk factors which contribute to cardiovascular disease.

## Contributors

AC, IK, PP and EM conceived the study. PP and ZT conducted analyses with input from AC, IK, and EM. AC drafted the initial version of the manuscript. All authors contributed to the interpretation of data, revision of the manuscript, and have approved the final version.

## Data sharing statement

Underlying data can be accessed from the WHO Mortality database:

https://www.who.int/data/data-collection-tools/who-mortality-database.

## Declaration of interests

ZT reports a previous summer internship with Novo Nordisk. MM reports consulting fees from Abbott, Cadrenal, Paragonix, Second Heart Assist, Medtronic, Janssen, Natera, and Moderna, and participation on the advisory boards of Mesoblast, Fineheart, Leviticus, and NupulseCV.
