# Supplementary material for: Trends in avoidable mortality from cardiovascular diseases in the European Union, 1995–2020: a retrospective secondary data analysis
Source: Lancet Reg Health Eur. 2024 Sep 27;47:101079. doi: 10.1016/j.lanepe.2024.101079 (PMC11470399; doi:10.1016/j.lanepe.2024.101079)
Supplement: Supplementary Material [file mmc1.pdf]

Supplementary Material

Table 1: Data availability per country 2

Table 2: Change in avoidable, preventable, and treatable cardiovascular mortality per country by sex 3

Figure 1: Avoidable cardiovascular mortality per country by cause, earliest and latest year 6

Figure 2: Joinpoint trends in a) avoidable cardiovascular mortality and b) by causes of death .. 7

Figure 3: Trends in potential years of life lost by sex and region, 1995-2019 8

Table 3: Sensitivity Analysis – Missing Years Imputation.. 9

Table 4: 2021 Avoidable cardiovascular mortality estimates..10

Table 1: Data availability per country

Country	Region	First year	Last year	Total years	Missing years	Data Usability*
Austria	Western	1995	2020	26		High
Belgium	Western	1995	2020	26		High
Bulgaria	Eastern	1995	2020	26		Low
Croatia	Eastern	1995	2020	26		High
Cyprus	Western	2004	2020	17	1995-2003	High
Czech Republic	Eastern	1995	2020	26		High
Denmark	Western	1995	2020	26		High
Estonia	Eastern	1995	2020	26		High
Finland	Western	1995	2020	26		High
France	Western	1995	2020	26		Medium
Germany	Western	1995	2020	26		High
Greece	Western	1995	2020	26		Medium
Hungary	Eastern	1995	2020	26		High
Ireland	Western	1995	2020	26		High
Italy	Western	1995	2020	26		High
Latvia	Eastern	1995	2020	26		High
Lithuania	Eastern	1995	2020	26		High
Luxembourg	Western	1995	2020	26		High
Malta	Western	1995	2020	26		High
Netherlands	Western	1995	2020	26		High
Poland	Eastern	1995	2020	24	1997,1998	Medium
Portugal	Western	1995	2019	22	2004-2006, 2020	High
Romania	Eastern	1995	2019	25	2020	High
Slovakia	Eastern	1995	2020	24	2011, 2015	High
Slovenia	Eastern	1995	2020	26		High
Spain	Western	1995	2020	26		High
Sweden	Western	1995	2020	26		High
United Kingdom	Western	1995	2020	25	2000	High

*Data useability is based on WHO assessment of data completeness and proportion of ill-defined or non-specific causes. Source: https://platform.who.int/mortality/about/data-quality

Table 2: Change in avoidable, preventable, and treatable cardiovascular mortality per country by sex

Country	Year	Male (ASMR per 100,000)			Female (ASMR per 100,000)		
		Avoidable	Preventable	Treatable	Avoidable	Preventable	Treatable
Austria	1995	240.79	115.91	124.88	101.62	46.52	55.10
	2020	87.91	42.47	45.44	34.95	16.29	18.66
	Annual change (%)	-3.95	-3.94	-3.96	-4.18	-4.11	-4.24
Belgium	1995	154.32	75.33	78.99	66.66	31.31	35.36
	2020	52.91	24.96	27.96	22.69	10.17	12.52
	Annual change (%)	-4.19	-4.32	-4.07	-4.22	-4.40	-4.07
Bulgaria	1995	503.31	249.09	254.21	266.35	130.07	136.28
	2020	335.42	158.37	177.05	139.51	65.32	74.19
	Annual change (%)	-1.61	-1.80	-1.44	-2.55	-2.72	-2.40
Croatia	1995	312.39	149.87	162.52	165.73	77.96	87.77
	2020	166.40	81.79	84.61	61.64	29.90	31.74
	Annual change (%)	-2.49	-2.39	-2.58	-3.88	-3.76	-3.99
Cyprus	2004	145.23	71.00	74.23	57.87	27.09	30.78
	2020	81.99	39.89	42.09	29.31	14.39	14.92
	Annual change (%)	-3.51	-3.54	-3.48	-4.16	-3.88	-4.43
Czech Republic	1995	471.84	225.84	245.99	216.94	100.51	116.42
	2020	152.70	72.43	80.27	55.34	24.94	30.40
	Annual change (%)	-4.41	-4.45	-4.38	-5.32	-5.42	-5.23
Denmark	1995	232.62	113.64	118.97	102.67	48.66	54.01
	2020	58.33	28.04	30.30	24.25	11.52	12.72
	Annual change (%)	-5.38	-5.44	-5.32	-5.61	-5.60	-5.62
Estonia	1995	687.48	337.98	349.51	294.20	143.65	150.55
	2020	209.20	100.68	108.52	66.35	30.73	35.62
	Annual change (%)	-4.65	-4.73	-4.57	-5.78	-5.98	-5.60
Finland	1995	304.68	151.16	153.51	103.81	50.81	53.00
	2020	109.32	53.51	55.81	35.73	16.68	19.05
	Annual change (%)	-4.02	-4.07	-3.97	-4.18	-4.36	-4.01
France	1995	113.19	53.87	59.32	39.55	17.76	21.79
	2020	46.20	21.88	24.32	16.63	7.41	9.22
	Annual change (%)	-3.52	-3.54	-3.50	-3.41	-3.44	-3.38
Germany	1995	235.27	113.10	122.17	98.64	45.37	53.27
	2020	91.43	43.14	48.29	35.43	16.00	19.43
	Annual change (%)	-3.71	-3.78	-3.64	-4.01	-4.08	-3.95

Country	Year	Male (ASMR per 100,000)			Female (ASMR per 100,000)		
		Avoidable	Preventable	Treatable	Avoidable	Preventable	Treatable
Greece	1995	190.85	93.73	97.12	91.87	44.30	47.57
	2020	119.08	58.42	60.66	39.81	18.98	20.83
	Annual change (%)	-1.87	-1.87	-1.87	-3.29	-3.33	-3.25
Hungary	1995	530.78	257.08	273.70	248.55	117.41	131.15
	2020	286.51	140.84	145.67	118.34	57.94	60.40
	Annual change (%)	-2.44	-2.38	-2.49	-2.92	-2.79	-3.05
Ireland	1995	318.99	156.90	162.09	134.29	64.06	70.23
	2020	75.62	36.33	39.29	27.62	12.61	15.02
	Annual change (%)	-5.59	-5.68	-5.51	-6.13	-6.30	-5.98
Italy	1995	159.14	77.67	81.47	68.22	31.93	36.29
	2020	63.09	30.71	32.37	25.55	12.03	13.52
	Annual change (%)	-3.63	-3.64	-3.62	-3.85	-3.83	-3.87
Latvia	1995	529.92	259.18	270.74	178.38	83.55	94.83
	2020	347.89	169.36	178.53	113.69	54.61	59.08
	Annual change (%)	-1.67	-1.69	-1.65	-1.79	-1.69	-1.87
Lithuania	1995	535.27	262.04	273.23	239.78	114.56	125.22
	2020	380.23	185.05	195.18	120.74	57.65	63.09
	Annual change (%)	-1.36	-1.38	-1.34	-2.71	-2.71	-2.70
Luxembourg	1995	174.78	86.36	88.42	75.31	37.32	37.99
	2020	55.51	24.54	30.96	25.19	11.33	13.86
	Annual change (%)	-4.48	-4.91	-4.11	-4.29	-4.66	-3.95
Malta	1995	210.42	104.23	106.19	129.79	62.97	66.82
	2020	93.80	44.76	49.04	34.07	15.39	18.67
	Annual change (%)	-3.18	-3.32	-3.04	-5.21	-5.48	-4.97
Netherlands	1995	192.59	94.62	97.98	77.81	37.41	40.40
	2020	45.00	21.78	23.22	22.35	10.51	11.84
	Annual change (%)	-4.87	-4.95	-4.79	-4.87	-4.95	-4.79
Poland	1995	349.91	166.52	183.39	163.47	75.77	87.70
	2020	180.08	87.73	92.35	64.81	31.16	33.65
	Annual change (%)	-2.62	-2.53	-2.71	-3.63	-3.49	-3.76
Portugal	1995	207.64	100.39	107.25	104.42	49.27	55.15
	2019	80.08	38.45	41.63	28.99	13.09	15.90
	Annual change (%)	-3.89	-3.92	-3.87	-5.20	-5.37	-5.05
Romania	1995	533.36	261.01	272.35	316.31	153.42	162.89
	2019	303.63	149.02	154.61	130.51	63.52	67.00

Country	Year	Male (ASMR per 100,000)			Female (ASMR per 100,000)		
		Avoidable	Preventable	Treatable	Avoidable	Preventable	Treatable
	Annual change (%)	-2.32	-2.31	-2.33	-3.62	-3.61	-3.63
Slovakia	1995	509.86	242.68	267.18	251.84	116.83	135.02
	2020	230.80	111.42	119.38	88.02	41.98	46.04
	Annual change (%)	-3.12	-3.07	-3.17	-4.12	-4.01	-4.21
Slovenia	1995	253.15	122.68	130.47	115.09	54.23	60.85
	2020	86.47	41.71	44.76	32.01	14.87	17.14
	Annual change (%)	-4.21	-4.22	-4.19	-4.99	-5.04	-4.94
Spain	1995	143.39	68.53	74.86	57.41	25.54	31.87
	2020	61.36	29.23	32.13	20.73	9.21	11.53
	Annual change (%)	-3.34	-3.35	-3.33	-3.99	-4.00	-3.99
Sweden	1995	219.11	106.65	112.46	81.43	37.99	43.44
	2020	65.45	31.37	34.08	27.70	13.21	14.49
	Annual change (%)	-4.72	-4.78	-4.66	-4.22	-4.14	-4.30
United Kingdom	1995	284.78	137.83	146.96	129.04	59.64	69.40
	2020	94.12	44.13	49.98	39.29	17.16	22.13
	Annual change (%)	-4.33	-4.45	-4.22	-4.64	-4.86	-4.47

Abbreviation: ASMR; age-standardized mortality rate.

Figure 1: Avoidable cardiovascular mortality per country by cause, earliest and latest year

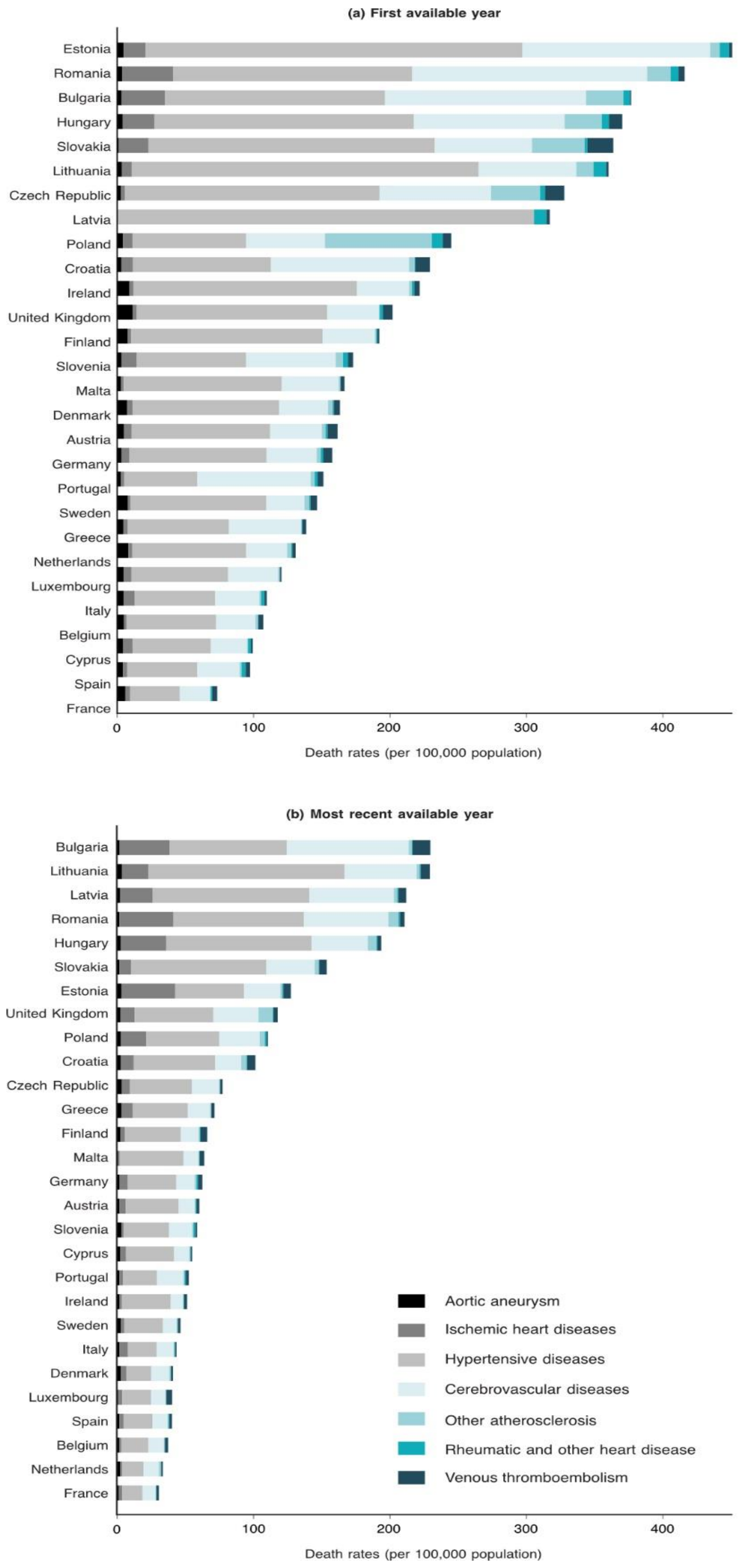

Figure 2: Joinpoint trends in a) avoidable cardiovascular mortality and b) by causes of death

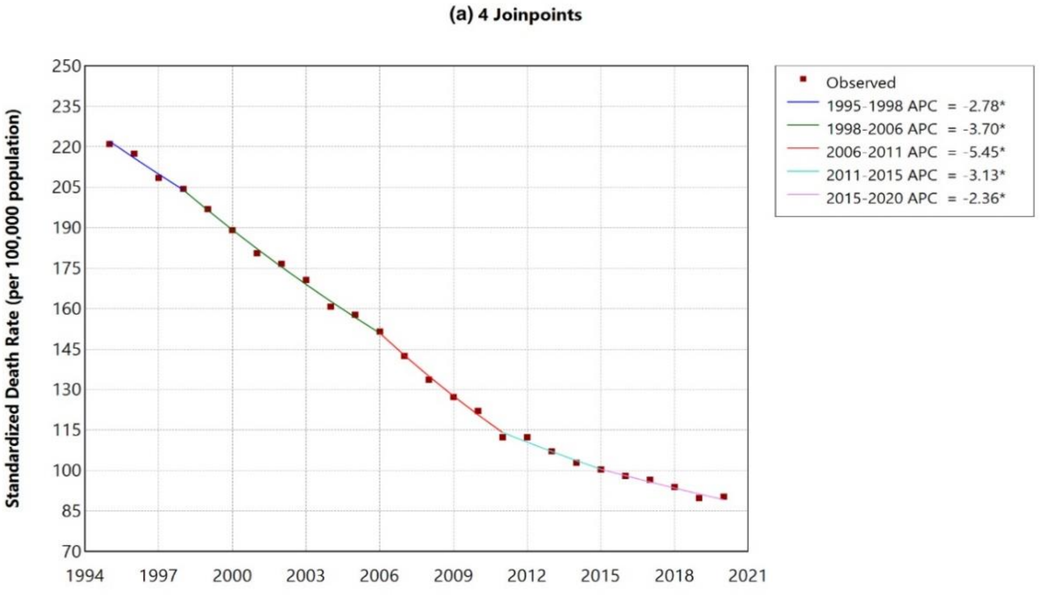

* Indicates that the Annual Percent Change (APC) is significantly different from zero at the alpha = 0.05 level.
-- Test Statistic and P-Value not available for the Empirical Quantile method.
Final Selected Model: 4 Joinpoints.

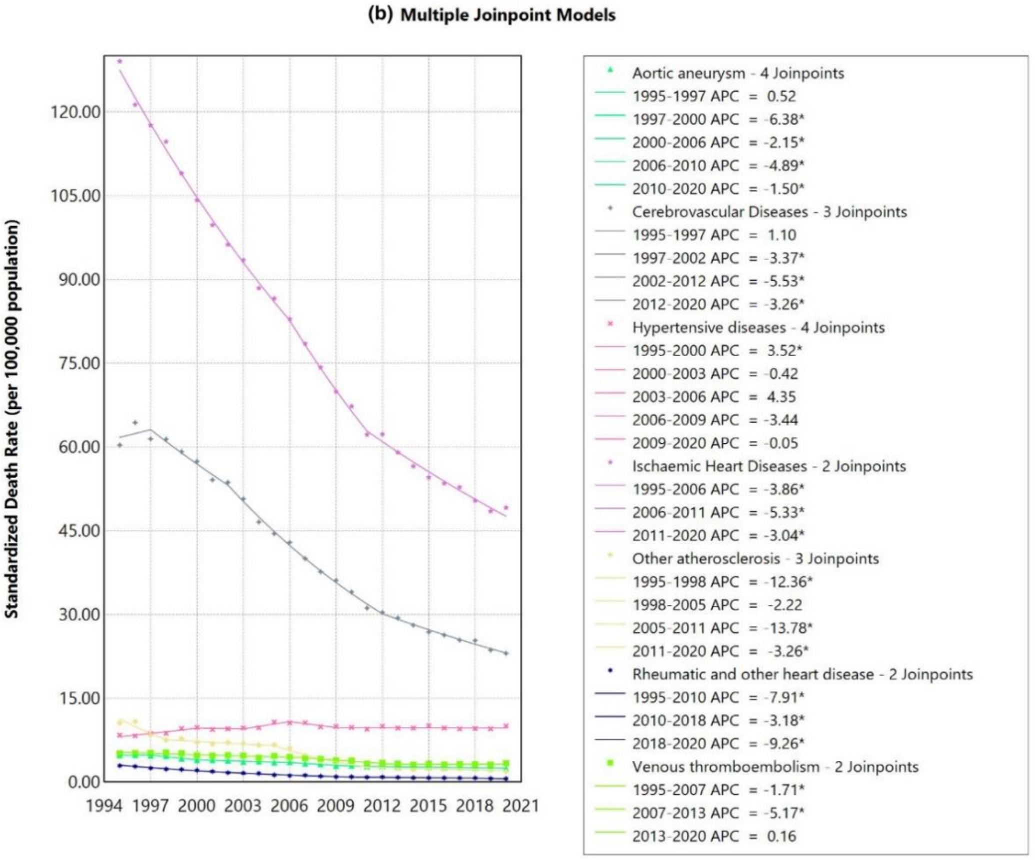

Figure 3: Trends in potential years of life lost by sex and region, 1995-2019

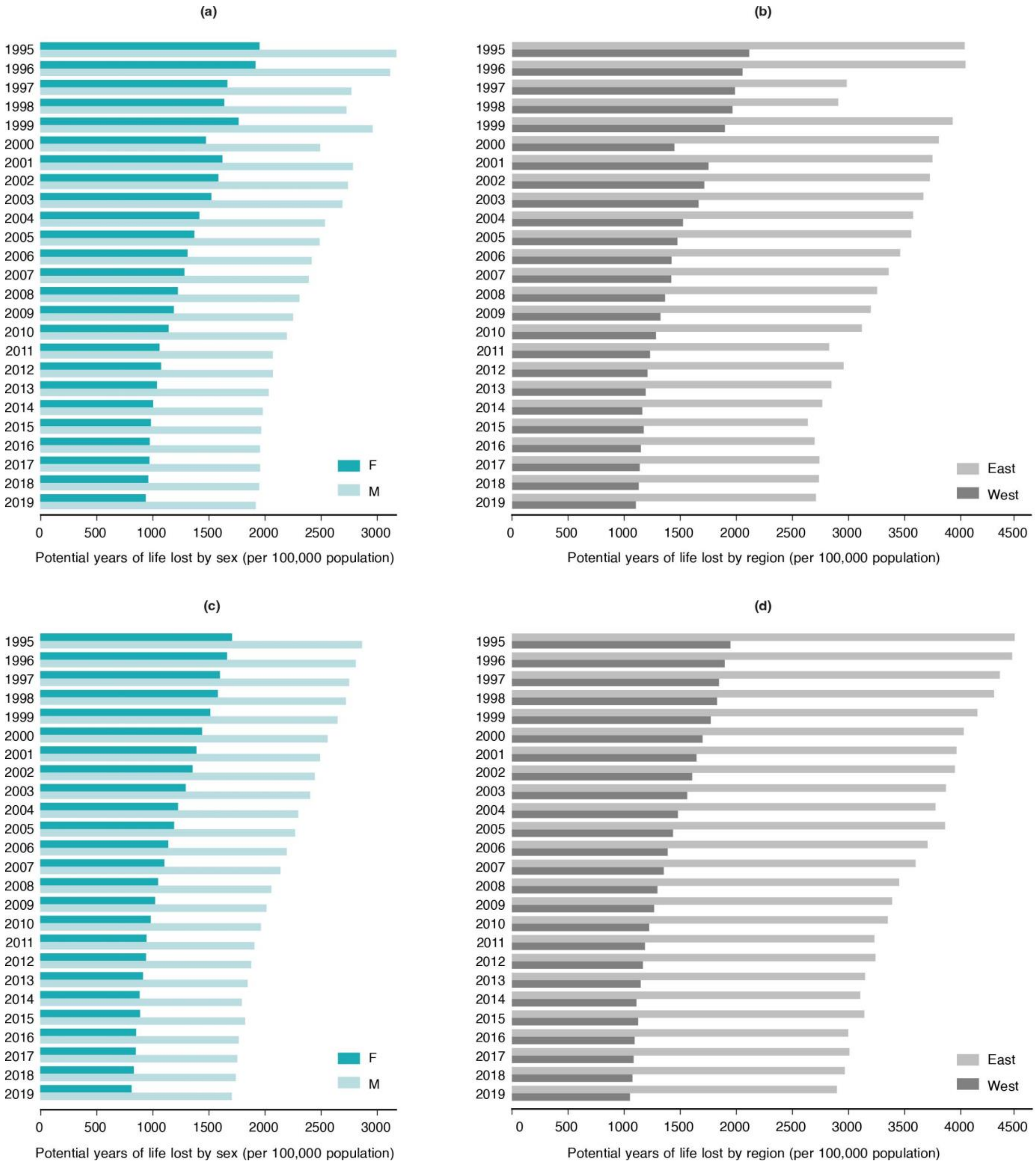

Note: Panels a and b correspond to the potential years of life years lost analysis reported in the manuscript (i.e. all available country-year observations). Panel c and d present a sensitivity analysis using data only from countries with a complete panel data series (i.e. countries without any missing yearly observations), which excludes Cyprus, Poland, Portugal, Romania, Slovakia, and the United Kingdom owing to gaps in data availability. Values are average age-standardized potential years of life lost.

Table 3: Sensitivity Analysis – Missing Years Imputation

Characteristics	Primary analysis	Linear interpolation		Last value carried forward	
		Value	% Difference	Value	% Difference
Disease					
All CVD	144.10	144.02	0.0%	144.33	0.2%
Aortic aneurysm	3.34	3.33	-0.3%	3.76	12.6%
Cerebrovascular disease	41.07	40.91	-0.4%	40.77	-0.7%
Hypertensive diseases	9.61	9.51	-1.0%	9.55	-0.6%
Ischemic heart diseases	79.70	78.94	-1.0%	78.69	-1.3%
Other atherosclerosis	4.98	5.02	0.8%	5.38	8.0%
Rheumatic and other heart disease	1.33	1.33	0.0%	1.34	0.7%
Venous thromboembolism	4.07	4.99	22.6%	4.94	21.4%
Sex					
Male	212.00	211.52	-0.2%	210.98	-0.5%
Female	89.62	89.81	0.2%	89.81	0.2%
Region					
Eastern Europe	233.10	232.69	-0.2%	232.74	-0.2%
Western Europe	85.68	86.78	1.3%	86.73	1.2%

Note: Estimates are age-standardized mortality rates per 100,000 except as otherwise noted.

Table 4: 2021 Avoidable cardiovascular mortality estimates

Country	Raw Mortality Figures			ASMR (per 100,000 population)		
	Male	Female	Total	Male	Female	Total
Austria	3,218	1,380	4,598	83.06	33.00	56.97
Bulgaria	11,497	6,154	17,651	351.37	150.45	242.09
Croatia	3,249	1,473	4,722	170.85	65.81	114.66
Cyprus	324	105	429	89.49	27.30	57.54
Czech Republic	7,341	3,219	10,560	148.77	56.10	99.84
Denmark	1,538	653	2,191	57.35	23.36	40.00
Estonia	1,130	523	1,653	213.47	71.90	132.90
Finland	2,965	1,095	4,060	106.67	36.11	70.16
Hungary	12,703	6,554	19,257	304.21	121.83	202.64
Latvia	2,870	1,342	4,212	385.11	125.03	233.23
Lithuania	4,027	1,759	5,786	370.90	115.09	221.59
Luxembourg	120	48	168	47.75	19.67	33.74
Netherlands	3,852	1,989	5,841	45.72	23.03	34.25
Poland	31,937	14,082	46,019	191.62	69.31	125.13
Slovakia	5,713	2,452	8,165	250.11	89.77	163.19
Spain	12,977	4,865	17,842	61.92	21.66	41.00
Sweden	2,924	1,203	4,127	63.90	25.48	44.55

Note: Mortality data for 2021 are provided for supplementary purposes. 2021 was not included in the full analysis owing to missing data for 11 countries.
